# Overexpression of fibulin-3 in tumor tissue predicts poor survival of malignant mesothelioma patients from hand-spinning asbestos exposed area in eastern China

**DOI:** 10.1038/s41598-020-77412-4

**Published:** 2020-11-23

**Authors:** Zhaoqiang Jiang, Wei Shen, Shibo Ying, Zhibin Gao, Xianglei He, Riping Chen, Hailing Xia, Xinnian Guo, Yuan Fang, Yixiao Zhang, Jianjiang Miao, Jian Zhou, Xing Zhang, Junqiang Chen, Jianlin Lou

**Affiliations:** 1 Institute of Occupational Diseases, Hangzhou Medical College (Zhejiang Academy of Medical Sciences), 182 Tianmushan Road, Hangzhou, 310013 Zhejiang People’s Republic of China; 2 The Third People’s Hospital of Cixi, Ningbo, People’s Republic of China; 3 Department of Pathology, Yuyao People’s Hospital, Ningbo, People’s Republic of China; 4 Department of Pathology, Zhejiang Provincial People’s Hospital, Hangzhou, People’s Republic of China; 5 Institute of Hygiene, Hangzhou Medical College (Zhejiang Academy of Medical Sciences), Hangzhou, People’s Republic of China

**Keywords:** Biomarkers, Oncology

## Abstract

Fibulin-3 is an extracellular matrix glycoprotein widely expressed in various tissues. Tissue fibulin-3 expression have never been reported in association with prognosis of mesothelioma. Hence, we sought to determine the association between fibulin-3 expression and mesothelioma survival. We made a tissue microarray, which was comprised of cancer and normal tissue from mesothelioma patients (*n* = 82) during the period 1998–2017 in China. Fibulin-3 and HGMB1 expression were analyzed by immunohistochemistry method. Kaplan–Meier method and Cox proportional hazard models were used for analyzing survival data. Overall, 61 cases (74.4%) were female; 90.2% were of epithelioid type; the median overall survival time was 12.5 months. Fibulin-3 and HMGB1 were highly expressed in tumor tissue rather than adjacent tissue. The expression of fibulin-3 in tissue was correlated with that of HMGB1 (*r* = 0.32, *P* = 0.003). High expression of fibulin-3 in tumor tissue could predict poor survival in patients with mesothelioma (*P* = 0.02). This remained true in a multivariate model, with a significant hazard ratio of 1.91. We demonstrated that fibulin-3 in tumor tissue was a novel biomarker of poor survival of mesothelioma, suggesting it may be a relevant target for therapeutic intervention.

## Introduction

Malignant mesothelioma is a rare malignancy, predominantly arising from the pleura, along with the peritoneum, tunica vaginalis and pericardium^1^. It is mainly caused by the inhalation of asbestos fibers^2^. The incidence of mesothelioma has been increasing significantly with an average number of 40,000 deaths per year worldwide^3^. The increasing incidence rate of mesothelioma was found between 2000 and 2013 in China, with a peak crude rate of 3.49 per million in 2012^4^. How, in China and other developing countries where the use of asbestos have not been fully banned^5^, the reported incidence of malignant mesothelioma might be underestimated^6^. Until recently, a study^7^ in eastern China, including 92 mesothelioma patients, reported a unique fact of high peritoneal/pleural ratio and female/male ratio, suggesting that more attention should be paid on this deadly disease in China.

Chemotherapy, radiotherapy and surgery are the main methods for the treatment of mesothelioma, which can provide better survival in a part of mesothelioma patients^8^. However, as mesothelioma was always difficult to be diagnosed at early stage, approximately 50% of the mesothelioma patients died 4–16 months after diagnosis^9^. Thus, it is an urgent need to find more prognostic biomarkers for guiding treatment and prolonging survival time of mesothelioma patients. Tumor size, grade, and lymph node status were correlated with prognosis for patients of mesothelioma. Clinical treatments with chemotherapy, surgery, radiation, and novel therapies have been associated with prolonged survival time in mesothelioma patients^10^. Previous studies^11,12^ found that BAP1 mutation and Ki-67 index > 5% were associated with prolonged survival time of malignant mesothelioma, based on tissue microarray (TMA) method. However, the finding of novel prognostic biomarkers of mesothelioma is still needed.

Epidermal growth factor-containing fibulin-like extracellular matrix protein (EFEMP1), also called fibulin-3, has the function in suppressing tumor growth and angiogenesis, while promoting tumor cell invasion. Fibulin-3 expression varied in different kind of tumors. En-lin et al.^13^ found that overexpression of fibulin-3 in tissue was associated with poor survival in cervical carcinoma. Conversely, low expression of fibulin-3 in tissue was correlated with poor survival in hepatocellular carcinoma^14^ and colorectal cancer^15^. A previous study in the USA^16^ found that fibulin-3 level in plasma and effusion was independently predictive of overall survival of mesothelioma patients. Furthermore, the prognostic value of fibulin-3 was confirmed in a recent study^17^ with 114 patients of mesothelioma, which found that lower level of fibulin-3 in pleural effusion was associated with longer survival time. However, the link between tissue expression of fibulin-3 and overall survival for mesothelioma is not clear yet.

High mobility group box 1 (HMGB1) is a chromatin structural protein expressed in the nuclei of mammalian cells. It can stabilize nucleosomes and engage in DNA transcription, replication, and recombination^18^. The expression of HMGB1 served as a novel diagnostic biomarker in serum samples of mesothelioma^19^. More recently, a study^20^ of 170 patients with mesothelioma found that total immunohistochemistry score of HMGB1 in tissue was a useful prognostic biomarker for mesothelioma. However, the prognostic role of HMGB1 in nucleus still needed more clinical validation.

To date, the prognostic biomarkers of mesothelioma have not been fully studied yet. Therefore, in the present study, we first analyzed the expression of fibulin-3 and HMGB1 in tissues, using immunohistochemical evaluation method. Then we sought to characterize the link between fibulin-3 and HMGB1 expression and death outcome of patients with mesothelioma.

## Results

### Demographic and clinicopathological findings of mesothelioma patients

Mesothelioma patients were included between 1998 and 2017. At the end of this study, 12 cases (14.6%) were alive, and the median survival time was 12.5 months (95% *CI*: 10.5–18). The mean age at diagnosis was (57.5 ± 11.8) years, ranging from 36 to 86 years. Among 82 patients, 74.4% were female. Epithelioid subtype was found in 90.2% of patients. The proportion of peritoneal mesothelioma patients was 62.2%, while the proportion of patients undergoing surgery was 72.4%. Only 27 people were investigated for the history of asbestos exposure, and 92.6% of them were occupationally exposed to asbestos. Subsequent subgroup analysis showed that there was no significant difference in overall survival among different subgroups (Table 1).

**Table 1:** Demographics of mesothelioma patients.

Variables	*n* (%)	Median OS (months)	95% *CI*	χ^2^	*P*
Overall survival	82 (100)	12.5	10.5–18.0	–	–
**Gender**					
Male	21 (25.6)	10.5	9.5–26	0.0	0.886
Female	61 (74.4)	14.0	11–19.5		
**Age**					
< 60 years	50 (61.0)	15	12–21	2.9	0.089
≥ 60 years	32 (39.0)	10.5	7.3–13		
**Subtype**					
Epithelioid	74 (90.2)	12.5	10–18	0.0	0.879
Non-epithelioid^a^	8 (9.8)	10.5	9.6–26.8		
**Site**					
Pleural	31 (37.8)	12	9.5–21	0.1	0.748
Peritoneal	51 (62.2)	14.3	8–19.5		
**Treatment**					
Surgery^b^	63 (72.4)	12.5	9.5–18	0.2	0.640
Non-surgery^c^	24 (27.6)	12	10.5–24		

OS: overall survival.

^a^Including the patients with biphasic or sarcomatoid subtype.

^b^Including the patients undergoing chemotherapy after surgery.

^c^Including the patients undergoing chemotherapy, radiotherapy or no special treatment.

### Overexpression of fibulin-3 and HMGB1 in tumor tissue

**Figure 1:**
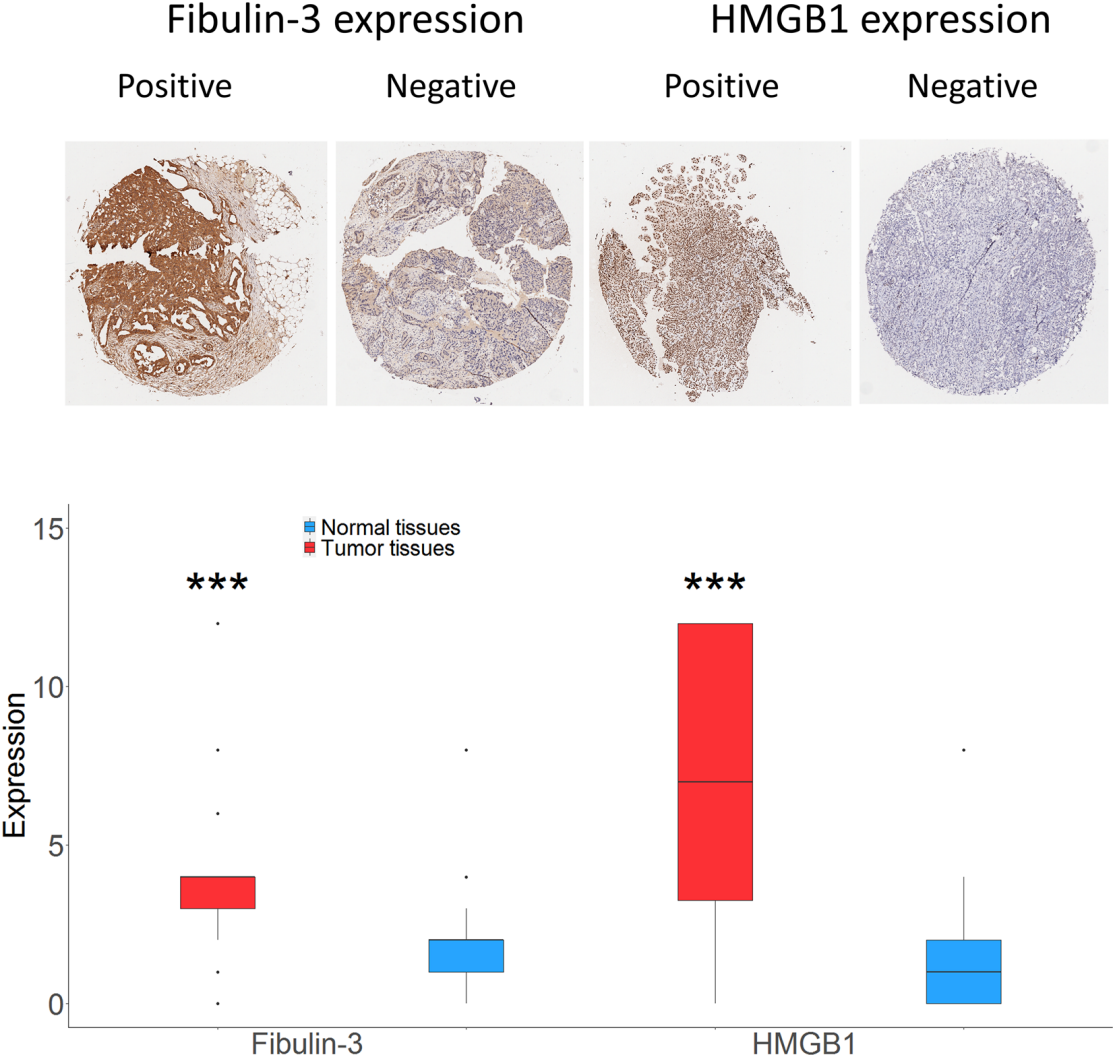
Fibulin-3 and HMGB1 expression between tumor tissues and adjacent tissues. The above photomicrographs illustrate the immunohistochemistry of fibulin-3 and HMGB1 expression; the magnification is 400. The below figure showed the expression of fibulin-3 and HMGB1 using staining scores; *** *P* < 0.001, compared with adjacent tissues.

Representative high and low expression of fibulin-3 and HMGB1 in tissue were shown in Fig. 1. Generally, positive expression of fibulin-3 was detected in the cytoplasm, while the expression of HMGB1 protein was commonly observed in the nuclear of tumor cell (Fig. 1). Adjacent tissues showed a weak expression of fibulin-3 and HMGB1 with median expressions of 2 (1–2) and 4 (1–6), respectively. However, tumor tissues showed a higher expression of fibulin-3 and HMGB1 with median expressions of 4.0 (3–4) and 7 (3.3–12). The tumor tissues showed a higher expression of fibulin-3 and HMGB1 than adjacent tissues (*V* = 1659.5 and 1706, respectively; *P* < 0.001).

### Fibulin-3 and HMGB1 expression are not different between subgroups

**Figure 2:**
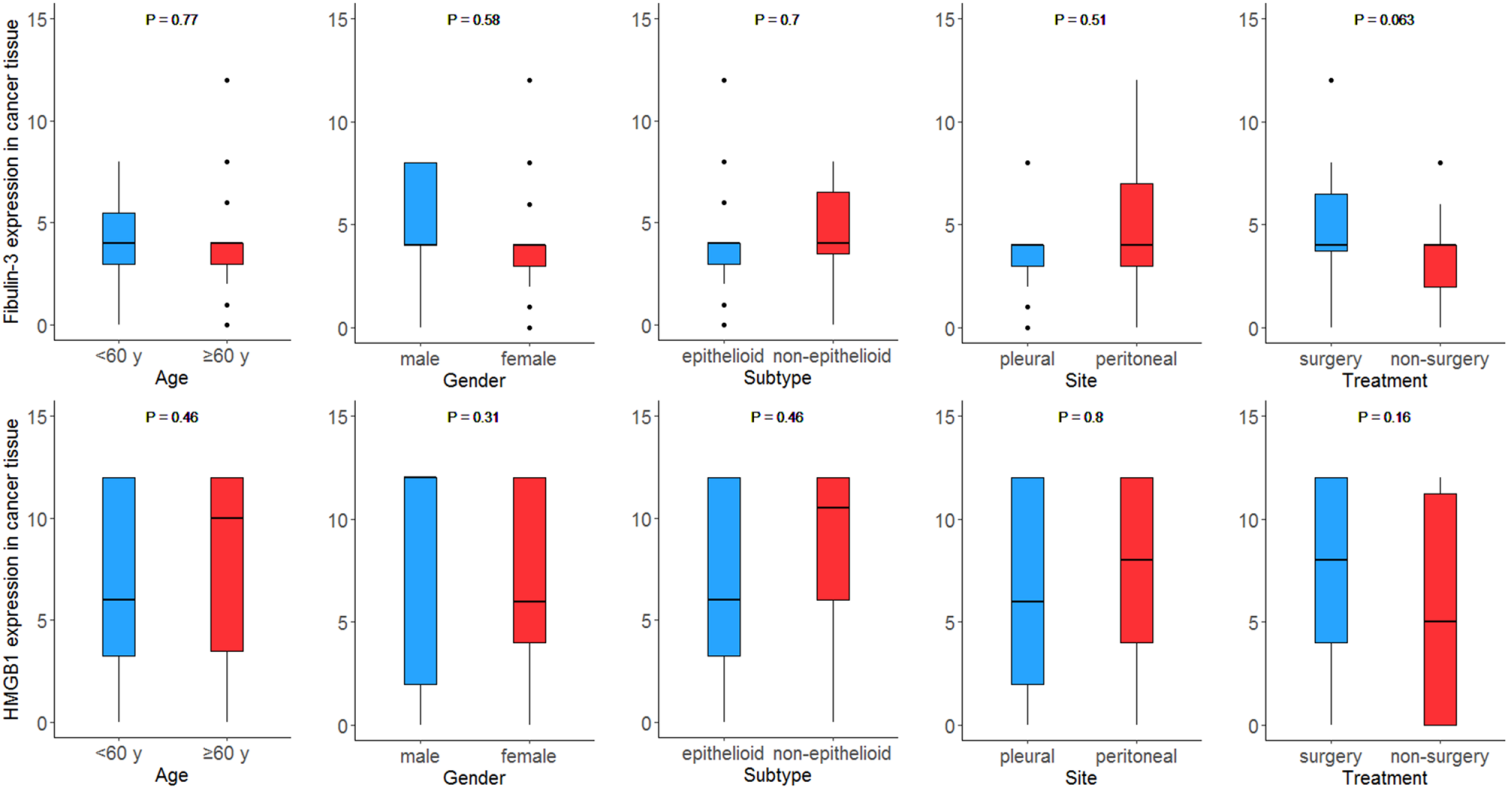
Expressional differences of Fibulin-3 and HMGB1 in different gender, age, type, site, and treatment methods. (**A**) Fibulin-3 positive expression in tissue across different age. (**B**) Fibulin-3 positive expression in tissue across different gender. (**C**) Fibulin-3 positive expression in tissue across different type. (**D**) Fibulin-3 positive expression in tissue across different site. (**E**) Fibulin-3 positive expression in tissue across different treatment. (**F**) HMGB1 positive expression in tissue across different age. (**G**) HMGB1 positive expression in tissue across different gender. (**H**) HMGB1 positive expression in tissue across different type. (**I**) HMGB1 positive expression in tissue across different site. (**J**) HMGB1 positive expression in tissue across different treatment.

Among all the mesothelioma patients, the median expression of fibulin-3 and HMGB1 was 4 (3–4) and 7 (3–12), separately (Fig. 2). There were 20 mesothelioma patients (24.4%) with fibulin-3 high expression and 41 patients (50%) with HMGB1 high expression. The median expression of fibulin-3 was the same among the patients in each subgroup (median expression = 4). HMGB1 was higher expressed in the patients of non-epithelioid type, with a median expression of 10.5 compared to those of epithelioid type (median expression = 6). Median expression of HGMB1 was 8 among patients of peritoneal mesothelioma and those with surgery treatment, while the median expression was 6 in patients of pleural mesothelioma and 5 in those with non-surgery treatment. However, HMGB1 or fibulin-3 expression was not significantly related to age, gender, subtype, site, or treatment method (*P* > 0.05).

### Fibulin-3 and HMGB1 expressions are partially correlated across clinicopathological variables

**Figure 3:**
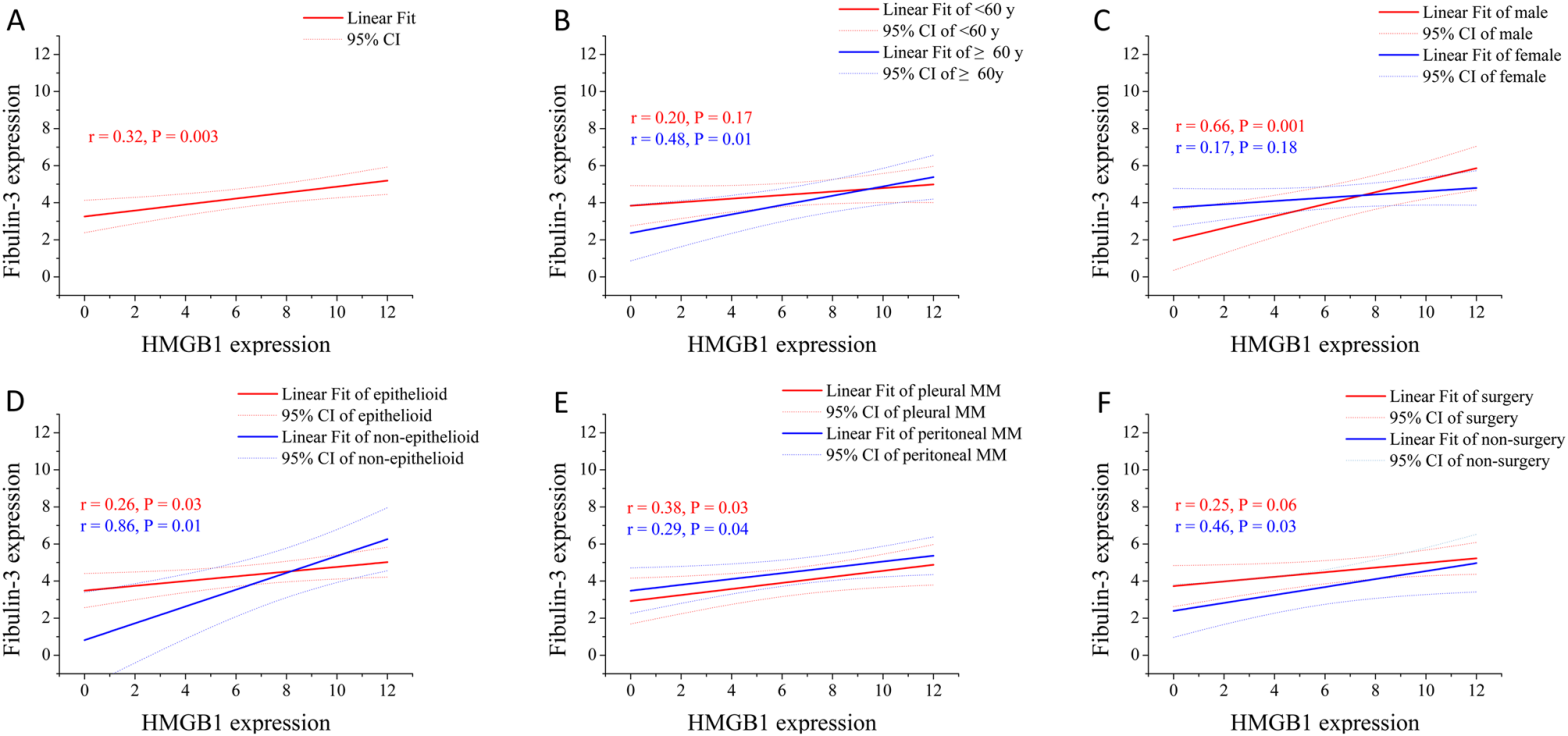
Fitting lines and 95% confidence intervals of fibulin-3 and HMGB1 expression. (**A**) Overall fitting line and 95% confidence intervals between fibulin-3 and HMGB1 expression. (**B**) Fitting line and 95% confidence intervals between fibulin-3 and HMGB1 expression in different genders. (**C**) Fitting line and 95% confidence intervals between fibulin-3 and HMGB1 expression in different ages. (**D**) Fitting line and 95% confidence intervals between fibulin-3 and HMGB1 expression in different subtypes. (**E**) Fitting line and 95% confidence intervals between fibulin-3 and HMGB1 expression in different sites. (**F**) Fitting line and 95% confidence intervals between fibulin-3 and HMGB1 expression in different treatments.

For all the patients in the study, the expression of fibulin-3 in tissue was significantly correlated with that of HMGB1 (*r* = 0.32, *P* = 0.003; Fig. 3). The relationship between fibulin-3 and HMGB1 expression was significant among male patients, those of ≥ 60 years, and those with non-surgery treatment (*r* = 0.66, 0.48, and 0.46, *P* = 0.001, 0.01, and 0.03, respectively), while that was not significant in female patients, those of < 60 years, and those with surgery treatment (*P* > 0.05). The correlation coefficient between fibulin-3 and HMGB1 expression for non-epithelioid type was very high (*r* = 0.86, *P* = 0.01), while that for epithelioid type was only 0.26 (*P* = 0.03). The correlation coefficients for pleural and peritoneal mesothelioma did not vary much (*r* = 0.39 and 0.29, *P* = 0.03 and 0.04, respectively).

### High fibulin-3 expression is associated with worse outcome

**Figure 4:**
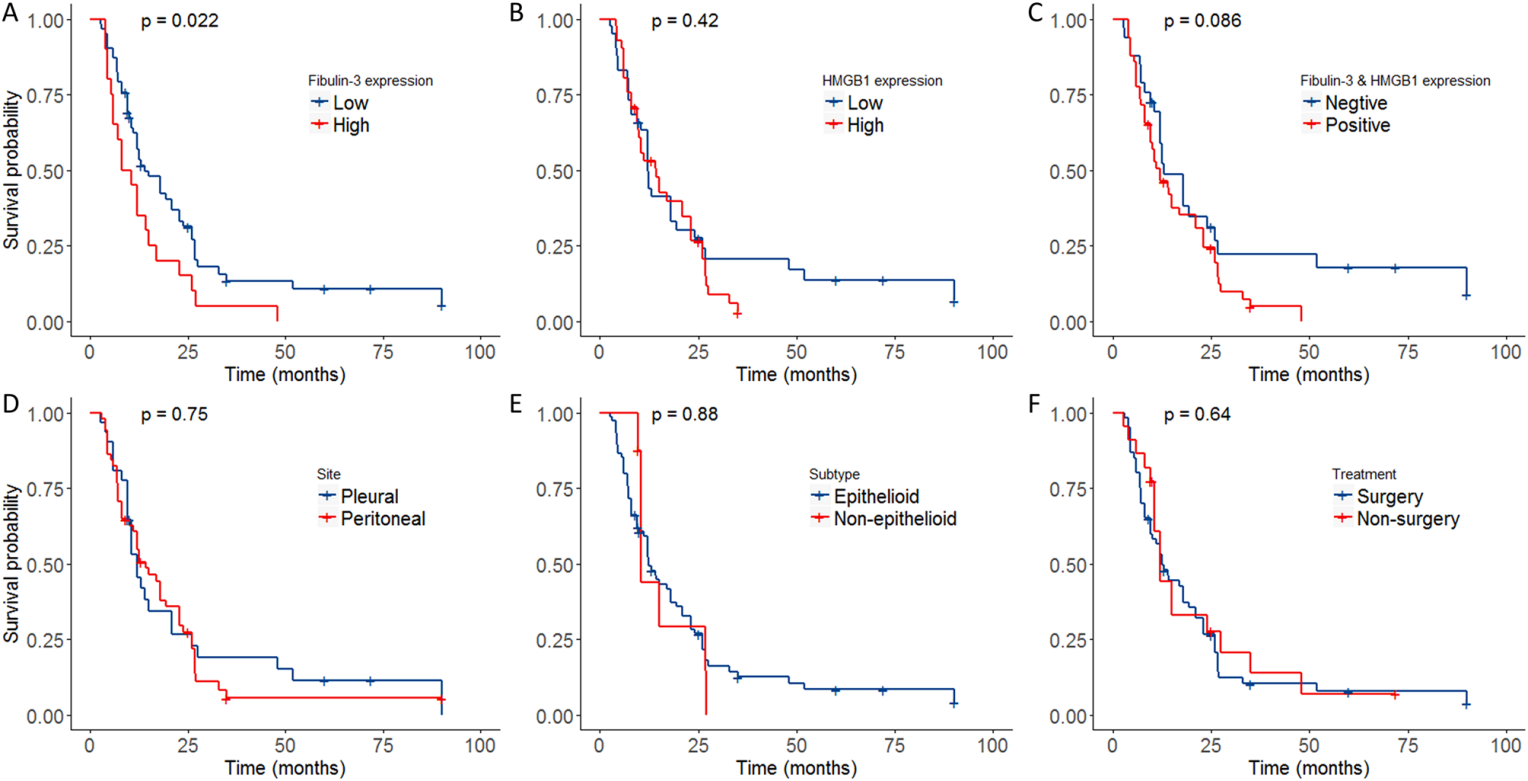
Survival plots of fibulin-3 and HMGB1 classified by tumor sites and tumor type. (**A**) Overall survival rate of mesothelioma patients. (**B**) Survival plot of different gender of mesothelioma patients. (**C**) Survival plot of different age of mesothelioma patients. (**D**) Survival plot of different histological subtype of mesothelioma patients. (**E**) Survival plot of different site of mesothelioma patients. (**F**) Survival plot of different treatment of mesothelioma patients. (**G**) Survival plot of different fibulin-3 expression of mesothelioma patients. (**H**) Survival plot of different HMGB1 expression of mesothelioma patients.

Figure 4 showed overall survival curve across different levels of fibulin-3 expression, HMGB1 expression, combination of fibulin-3 and HMGB1, treatment method, and subtype. Higher expression of fibulin-3 was correlated with poor survival (*P* = 0.02; Fig. 4A). The median survival time of patients with low fibulin-3 expression was 14 months, while that of patients with high fibulin-3 expression was 9.3 months. HMGB1 expression was not associated with poor survival of mesothelioma (*P* = 0.42, Fig. 4B). When combined HMGB1 with fibulin-3 expression, patients with positive expression had longer survival time than those with co-negative expression (14 vs. 12.5 months). However, the combination expression of HMGB1 and fibulin-3 was not related to the overall survival of mesothelioma (*P* = 0.09; Fig. 4C). Meanwhile, subtype, site, or treatment method was not significantly associated with overall survival of mesothelioma (*P* > 0.05; Fig. 4D–F).

### Fibulin-3 overexpression is an independent prognostic factor for the survival rate of mesothelioma

High expression of fibulin-3 in tissue could independently predict worse overall survival of mesothelioma (*HR* = 1.91, *P* = 0.02; model 1 of Table 2). However, HMGB1 was not correlated with overall survival of mesothelioma (*HR* = 1.22, *P* = 0.47, model 2 of Table 2). The combination expression of fibulin-3 and HMGB1 was not associated with overall survival (*HR* = 1.67, *P* = 0.06; model 3 of Table 2). Age was slightly correlated with poor survival of mesothelioma in three models (*HR* = 1.69, 1.73, and 1.02; *P* = 0.06, 0.05, and 0.06, respectively).

**Table 2:** Multiple cox regression models of overall survival in mesothelioma patients.

Variables	Model 1 (including fibulin-3)				Model 2 (including HMGB1)				Model 3 (including combination of fibulin-3 and HMGB1)			
	HR	95% CI	Z	*P*	HR	95% CI	Z	*P*	HR	95% CI	Z	*P*
Gender: female versus male	1.18	0.65–2.15	0.54	0.59	1.11	0.61–2.02	0.34	0.73	1.07	0.59–1.93	0.22	0.83
Age: ≥ 60 y ears versus < 60 years	1.69	0.98–2.91	1.89	0.06	1.73	1.00–2.98	1.96	0.05	1.02	1.00–1.04	1.88	0.06
Subtype: non-epithelioid versus epithelioid	0.70	0.28–1.75	− 0.76	0.45	0.81	0.33–1.99	− 0.46	0.65	0.83	0.35–1.96	− 0.42	0.67
Site: peritoneal versus pleural	1.08	0.63–1.86	0.27	0.79	1.12	0.64–1.93	0.39	0.70	0.97	0.57–1.67	-0.09	0.93
Treatment: non-surgery versus surgery	0.84	0.46–1.53	− 0.57	0.57	0.89	0.49–1.61	− 0.38	0.70	0.84	0.46–1.53	− 0.56	0.58
Fibulin-3 expression: high versus low	1.91	1.09–3.33	2.27	0.02	–	–	–	–	–	–	–	–
HMGB1 expression: high versus low	–	–	–	–	1.22	0.72–2.05	0.73	0.47	–	–	–	–
Combination of fibulin-3 and HMGB1: positive versus negative	–	–	–	–	–	–	–	–	1.67	0.98–2.85	1.87	0.06

HMGB1 was excluded from model 1, and fibulin-3 was excluded from model 2 due to collinearity between tissue expression of fibulin-3 and HMGB1.

*HR*: hazard ratio; *CI*: confidence interval.

### The association of fibulin-3 and prognosis of mesothelioma varies across subgroups

Subgroup analysis showed that fibulin-3 expression was significantly associated with prognosis in young people, women, patients with epithelial type, peritoneal mesothelioma patients, and surgical patients (*P* < 0.05; Table 3). In detail, mesothelioma patients with high expression of fibulin-3 had poor survival (median survival time: 8 vs. 19.5 months) than those with low expression among < 60 years patients. Compared with female cases of low expression of fibulin-3, those with high expression of fibulin-3 had a significant poor survival (median survival time = 8 months). High expression of fibulin-3 was also linked with poorer survival of mesothelioma among patients with epithelial type (median survival time = 10.5 months), peritoneal mesothelioma patients (median survival time = 8 months), and surgical patients (median survival time = 7.6 months). High expression of HMGB1 was significantly associated with poor survival (median survival time = 9.6 months), while high expression of fibulin-3 and HMGB1 combination was associated with poor survival among patients of pleural mesothelioma (median survival time = 10.5 months).

**Table 3:** Association between fibulin-3, HMGB1 expression and prognosis of mesothelioma within subgroups.

Testing index	Variable	Category	Survival time, months		χ^2^	*P*
			Low expression	High expression		
Fibulin-3	Age	< 60 years	19.5	8	7.4	**0.007**
		≥ 60 years	10.5	10.5	0.0	0.937
	Gender	Male	10.5	13.8	0.1	0.746
		Female	18	8	6.3	**0.012**
	Site	Pleural	13	10.5	0.8	0.369
		Peritoneal	18	8	5.2	**0.023**
	Type	Epithelioid	15	8	6.7	**0.010**
		Non-epithelioid	10.5	15	0.9	0.333
	Treatment	Surgery	18	7.6	7.0	**0.008**
		Non-surgery	12	13.5	0.1	0.801
HMGB1	Age	< 60 years	12.5	17	0.0	0.899
		≥ 60 years	11.2	9.6	1.3	0.257
	Gender	Male	13	10.5	1.1	0.299
		Female	12	15	0.1	0.801
	Site	Pleural	13	9.6	7.0	**0.008**
		Peritoneal	12	23	2.4	0.124
	Type	Epithelioid	12.5	14.3	0.6	0.441
		Non-epithelioid	10.5	12.8	0.1	0.705
	Treatment	Surgery	12.2	14.3	0.0	0.987
		Non-surgery	12	10.5	1.7	0.188
Fibulin-3 and HMGB1	Age	< 60 years	18	14.3	1.5	0.217
		≥ 60 years	12	9.5	2.0	0.162
	Gender	Male	13	10.5	1.1	0.299
		Female	18	13	1.8	0.185
	Site	Pleural	13	10.5	5.4	**0.021**
		Peritoneal	12.5	14.3	0.0	0.909
	Type	Epithelioid	13	12	3.0	0.084
		Non-epithelioid	10.5	12.8	0.1	0.705
	Treatment	Surgery	15.5	11	1.6	0.200
		Non-surgery	12	12	0.6	0.445

The bold *P* value was < 0.05.

## Discussion

Fibulin-3 expression in tissue has never been studied in the prognosis of malignant mesothelioma. To our knowledge, this is the first study in China which analyzes the association between tissue biomarkers and overall survival of mesothelioma patients, and 62.2% were peritoneal mesothelioma. We found overexpression of fibulin-3 and HMGB1 in tumor tissue compared with adjacent tissue. Notably, our results indicated that the overexpression of tissue fibulin-3 was significantly associated with poor survival in mesothelioma patients, indicating that fibulin-3 expression in tissue may be a valuable prognostic marker for mesothelioma patients.

Studies on prognostic factors for mesothelioma are important in terms of searching for target therapy and prolong the life of mesothelioma patients. Although many improvements have been made in the diagnosis and treatment in mesothelioma, mesothelioma still represents an important cause of mortality across the world^21^. The median survival time of mesothelioma patients was 12.5 months in our study, which was consistent with previous study in the same province in China^22^. Previous study^23^ has listed several prognostic factors of mesothelioma, such as performance score, tumor histology, stage, age and gender. However, we found gender, age, subtype, site, and treatment were not correlated with overall survival of mesothelioma patients, and similar results were found in other studies^24,25^. Although treatment was reported to be a significantly prognostic factor for the prognosis^26^, that study only compared the patients undergoing chemotherapy with those untreated.

Fibulin-3 is usually lowly expressed in adjacent tissue^27^, however, it is secreted in body fluids and overexpressed in mesothelioma^28^. We found that fibulin-3 was highly expressed in tumor tissues rather than adjacent tissues, which was contradictory to recent studies in which downregulation was found in cutaneous squamous cell carcinoma^29^, colorectal cancer^15^, and hepatocellular carcinoma^14^. This difference may be explained by cell proliferation in a context-specific pattern in fibulin gene family^30^. In our study, the overexpression of fibulin-3 indicated that fibulin-3 may participate in the development of mesothelioma. Our results also showed that there was an upregulation of HMGB1 in mesothelioma tissues, which was in accordance with previous study^20^. Moreover, recent evidence^31^ found a significant correlation between HMGB1 expression and tumor stage in the cytoplasm of mesothelioma cell. To sum up, we can draw a conclusion that the expression of fibulin-3 and HMGB1 can well distinguish tumor tissue from adjacent tissue.

We revealed that high expression of fibulin-3 in tissue was correlated with poor survival of mesothelioma patients. This finding was similar to previous study^32^, which reported that effusion fibulin-3 was an independently prognostic factor for overall survival among 82 mesothelioma patients (*HR* = 2.05, *P* = 0.005). But they did not report prognostic value of tissue fibulin-3. Then, our subgroup analysis identified new target population for tissue fibulin-3 application in the prognosis study of mesothelioma. Therefore, our study is able to attribute this variety to the etiology of mesothelioma incidences, providing scientific basis for resettling the proper role of fibulin-3 in the prediction of mesothelioma prognosis. Furthermore, the association between tissue fibulin-3 and poor survival of mesothelioma patients remained significant on multivariate analysis, suggesting that fibulin-3 overexpression was an independent prognostic factor of mesothelioma. The association between high expression of fibulin-3 and poor survival in mesothelioma may be due to that fibulin-3 overexpression was associated with malignant transformation of mesothelial cells following exposure to asbestos or asbestos-like fibers^33^. However, due to the small sample size in each subgroup, it is warranted to validate our results through a larger sample size.

We found HMGB1 expression in nucleus was not linked with poor survival in K–M curve, which is in consistent with the result of previous study^20^. Previous report^34^ showed elevated expression of HMGB1 and fibulin-3 in blood samples from mesothelioma patients. Although fibulin-3 and HMGB1 both played important roles in chronic inflammation and vascular remodeling after asbestos or other asbestos-like fiber exposure^35,36^, the molecular relationship between these two indicators has not been found yet. Hence, the underpinning relationship are intriguing and warrant further confirmation.

This study had a number of strengths and some limitations. To our knowledge, this is the first study focusing on the prognostic tissue biomarker for malignant mesothelioma in Chinese population. The overexpression of fibulin-3 and HMGB1 could be used to differentiate between tumor and adjacent tissues. Moreover, our study demonstrated that the overexpression of tissue fibulin-3 was significantly correlated with poor survival of mesothelioma patients. For clinical treatment, our results indicated that tissue fibulin-3 could be used as a valuable biomarker to predict poor survival of mesothelioma patients. One limitation of this study is that this study is based on small tissue samples in the TMA which may not be representative of the whole sample of tissue. However, we used a multicenter design with mesothelioma patients from four hospitals to reduce this bias.

In summary, our data demonstrated an association of poor survival with tissue fibulin-3 overexpression in mesothelioma patients for the first time. Fibulin-3 positive expression correlated with worse overall survival among patients of < 60 years, women, patients with epithelial type, peritoneal mesothelioma patients, and surgical patients. HMGB1 was overexpressed in tumor tissue, but not correlated with overall survival of mesothelioma. The findings also provided new insights into molecular mechanisms of prognosis of mesothelioma, and then lead to new strategy for effective treatment.

## Methods

### Study populations

All 82 mesothelioma cases from four hospitals were pathologically diagnosed. Tumor tissue samples were taken at the time of diagnostic biopsy or surgery before any treatment. A total of 82 formalin fixed paraffin embedded tissue blocks between 1998 and 2017 were retrospectively obtained for further immunohistochemical analysis. Clinical information was achieved from admission record. Survival status was obtained according to telephone follow-up. Overall survival was defined from the time of diagnosis to the time of death or censoring. The excluding criteria was: tissue sample loss; unavailability of survival data.

### Tissue microarray

Representative area with more than 50% of tumor cells were selected, and a tissue microarray (TMA) method was applied. Totally, 82 tumor tissue and 67 specimens of adjacent tissues were included in the study. Histology of mesothelioma was reviewed independently by three pathologists. The remaining 15 specimens of adjacent tissues were not interpretable due to loss of tissue on the TMA slide. TMA sections were used for hematoxylin-eosin and immunohistochemical staining. The location on the corresponding block was marked. Array wax blocks were made and punched using array instrument (Beecher, USA). Triplicate 1–1.5 mm cores were taken from formalin fixed paraffin embedded tissue blocks using a semi-automated system to generate tissue microarray. Continuous slices with thick 5 μm were made in both tumor tissue and adjacent tissue (negative control).

### IHC staining

Expression of fibulin-3 and HMGB1 in tumor site and adjacent site was evaluated using immunohistochemistry (IHC) staining, and a standard protocol was applied for the immunostaining. Microarray sections (5-μm) were baked in the oven for 1 h at 63 °C, then deparaffinised with xylene and rehydrated. Antigen retrieval was performed in high-pressure citrate buffer (PH = 6.0) for 5 min. Following incubation in 10% normal goat serum for 30 min at room temperature, the sections were stained with the primary antibodies at 1:100 dilutions overnight at 4 °C. The antibodies were monoclonal (rabbit antihuman) with fibulin-3 and HMGB1 (Abcam Inc., Cambridge, MA). The primary antibodies were examined using a horseradish peroxidase enzyme-labeled polymer conjugated to anti-rabbit secondary antibodies.

### Staining scores

The TMA slide was digitized by Aperio ScanScope XTslide scanner (Aperio Technologies, Vista, CA) at 20× magnification. The expression of fibulin-3 and HMGB1 was scored for the percentage of positive cells (i.e. staining extent) and the intensity of staining. Staining extent was classified as: 0 (< 1%), 1 (1–25%), 2 (26–50%), 3 (51–74%), and 4 (≥ 75%), whereas staining intensity was scored as: 0 (no staining), 1 (weak staining, detectable above background), 2 (moderate staining), and 3 (intense staining) . For HMGB1, nuclear staining was considered, while cytoplasmic staining was considered for fibulin-3. A comprehensive staining index was calculated by the product of staining extent and staining intensity, ranging from 0 to 12. A staining index above median value was considered as high expression, while that below median value was considered as low expression. Eventually, samples with a staining score of > 4 were considered as high expression of fibulin-3, whereas those with a staining score of > 7 were considered as high expression of HMGB1. Then, the combination of fibulin-3 and HMGB1 expression was calculated, in which low expression of both fibulin-3 and HMGB1 was considered as negative, and high expression was considered as positive.

### Statistical analysis

Quantitative data with normal distribution were reported as ($\overline{x} \pm s$), while data with non-normal distribution were presented as median (25th–75th percentile). Qualitative data were reported as frequency and proportion. Wilcoxon signed rank test was used to compare the expression between tumor and adjacent tissues among 67 patients. Pearson correlation coefficient was used to compare fibulin-3 expression with HMGB1 expression. Survival probability was detected using log-rank test and Kaplan–meier (K–M) method. Multivariate Cox proportional hazard models were used for analysis of survival data, and hazard ratios (*HRs*) and 95% confidence intervals (*CIs*) were assessed. Two-side *P* < 0.05 was considered to be statistically significant. Statistical analyses were preformed using R Studio (version 1.1.453).

### Ethical approval

All participants gave written informed consent. This study were carried out in accordance with the Declaration of Helsinki, and approved by Ethics Committee of Zhejiang Academy of Medical Sciences (Code No. 10; date of approval: August 8, 2018).

## Abbreviations

CI: Confidence interval
HRs: Hazard ratios
HMGB1: High mobility group box 1
K–M: Kaplan–Meier
SD: Standard deviation
TMA: Tissue microarray
